# Commentary: Internal medicine at the crossroads of long COVID diagnosis and management

**DOI:** 10.3389/fmed.2026.1798119

**Published:** 2026-04-15

**Authors:** Martin Spanoghe, Thomas H. J. Molmans, Tomaso Antonacci, Emilie Burel, Florence Herschke, Nicole Schneider, Pauline Oustric, Paul Thielemans, Jean-Baptiste Nicolas, Nicolas Dauby, Charles Nicaise, Johan Van Weyenbergh, Marc Jamoulle

**Affiliations:** 1 Long Covid Belgium, Patient Organization, Namur, Belgium; 2 Laboratoire de biotechnologie et biologie appliquée, Département d'Agrobiosciences et chimie, Haute Ecole Provinciale de Hainaut Condorcet, Ath, Belgium; 3 Post-COVID Network Netherlands, Leiden, Netherlands; 4 Stichting Long COVID/Dutch Foundation for Long COVID Research, Bosch en Duin, Netherlands; 5 Department of Research and Development, AMC Bio, Strasbourg, France; 6 Department of Psychology, FernUniversität, Hagen, Germany; 7 Association #ApresJ20 Covid Long France, Patient Organization, Paris, France; 8 Internal Medicine, Centre Hospitalier EpiCURA, Ath, Belgium; 9 Service de Médecine Interne Générale, Onco-Hématologie, Endocrinologie et Rhumatologie, Clinique Saint-Luc Bouge, Namur, Belgium; 10 Department of Infectious Diseases, CHU Saint-Pierre, Université Libre de Bruxelles (ULB), Brussels, Belgium; 11 School of Public Health, Université Libre de Bruxelles (ULB), Brussels, Belgium; 12 U-CRI, Université Libre de Bruxelles (ULB), Brussels, Belgium; 13 URPhyM, NARILIS, Université de Namur, Namur, Belgium; 14 Laboratory of Clinical and Epidemiological Virology, Department of Microbiology, Immunology and Transplantation, Rega Institute for Medical Research, KU Leuven, Leuven, Belgium; 15 Department of General Practice, and Business Management Systems, University of Liège (HEC–ULiège), Liège, Belgium

**Keywords:** biomedical evidence, clinical uncertainty, epistemic humility, long Covid, patient harm, post-acute infection syndromes, psychologization, reflexivity

## When psychologizing Long COVID causes harm

We read with attention the article by Ranque and Cogan (1) entitled “*Internal medicine at the crossroads of Long COVID diagnosis and management*.” While the authors raise questions regarding the interplay between persistent symptoms, psychological factors, and illness perception in Long COVID (LC), key aspects of their interpretation do not reflect substantial biomedical evidence, thereby affecting the conclusions drawn. In addition, little attention is paid to the epistemic and clinical implications of uncertainty, including the harm it has caused patients.^1^ This commentary offers a constructive critique and proposes a more comprehensive perspective of LC with direct impact on patient care.

## 1 Analytical framework

### 1.1 Methodological considerations and conceptual clarity

The authors adopt a narrative review approach rather than a systematic evaluation of evidence strength. In the absence of meta-analytic synthesis, risk-of-bias assessment, or standardized quality appraisal, causal inferences remain limited and prone to extrapolations (2, 3).

The article relies on broad and heterogeneous definitions of LC, conflating self-reported prolonged symptoms with clinically confirmed cases, while not consistently applying standardized criteria proposed by WHO, NASEM, or ISARIC (4, 5). Such criteria are essential for conceptual coherence, etiological interpretations, and the avoidance of overgeneralization (6). Similarly, their survey relies on a non-random, voluntary sample confined to a single national context, thereby limiting representativeness and introducing potential bias.

The discussion emphasizes that psychological factors drive and perpetuate LC symptoms through the theoretical and clinically applied construct of “Functional Somatic Disorder (FSD),” promoted as a replacement for earlier notions such as *somatoform disorders* or *medically unexplained symptoms* (7, 8). However, the existing literature is limited by selection bias and reliance on self-report screening tools, risking conflating primary pathology with psychological symptoms and obscuring causality. It has also not yielded mechanistic insight or objective outcomes and lacks longitudinal follow-up. Moreover, FSD has been criticized for its lack of conceptual and clinical robustness, including poor differentiation between overlapping patient groups, blurred diagnostic boundaries, and overly inclusive criteria, thereby functioning as a broad residual category rather than a clearly delineated disease entity (9–12). Without longitudinal or causal-modeling analyses, assigning primary etiological significance to psychological factors likely overlooks reverse causality and overstates their role (13, 14). In particular, citing the failure of biomedical interventions in clinical trials to support psychological explanations seems unwarranted, given that trial authors themselves acknowledged a lack of biomarkers as a key limitation (15). By contrast, no analogous conclusion is drawn from the limited evidence base for “*cognitive behavioral therapy*” and “*gradual physical activity*” (16, 17).

### 1.2 Scope and selection of evidence

Of further concern is the selective emphasis on functional or psychosomatic explanations, as reflected in three biomedical evidentiary gaps, constraining mechanistic interpretation and, in turn, the conclusions drawn:

**(i) Immunological uncertainty**

Claims that there is no consensus on immunological mechanisms are not well-supported when grounded in a restricted citation base rather than in a comprehensive appraisal of the literature (18), a standard not applied consistently to other post-acute infection syndromes (PAIS) such as Guillain-Barré (19).

**(ii) Pre- and post-pandemic multisystem mechanisms**

The authors do not consider pre-pandemic evidence linking human coronaviruses to prolonged symptoms (20–24), nor key post-pandemic research increasingly pointing to associations with viral persistence, immune dysregulation, neuro-inflammation, endothelial/microvascular and skeletal muscle damage, mitochondrial dysfunction, blood–brain barrier disruption, autonomic impairment, post-exertional malaise and broader multi-system injury (25–50). Emerging evidence of causal mechanisms further supports biologically grounded hypotheses and motivates mechanistic and interventional research (51).

**(iii) Neuroimaging, biomarkers, and replicated signals**

Advanced neuroimaging has reported neural and metabolic alterations, interpreted as consistent with diffuse gliosis, distinct from primary psychiatric disease, and aligned with LC patients' symptoms (52–55). In addition, candidate biomarkers provide converging support for the hypothesis of viral persistence replicated across different cohorts (56–58).

## 2 Impact of functional diagnoses on Long COVID patient wellbeing

LC is a complex condition whose persistent uncertainty places patients in a position of heightened vulnerability and clinicians in an epistemic bind. Sociological research in healthcare shows that clinical uncertainty is not merely epistemic but also relational and emotional, with “*not knowing*” itself being burdensome (59, 60). When clinicians fail to engage in reflexivity (e.g., acknowledging their own limitations, knowledge gaps, and the relational impact of uncertainty) and instead advance judgments, or at least suggestive assertions, that frame illness primarily or disproportionately as psychosomatic/psychosocial, patients may further experience avoidable distress, self-doubt, diminished agency, isolation, and further psychological burden (60–64).

**Figure 1:**
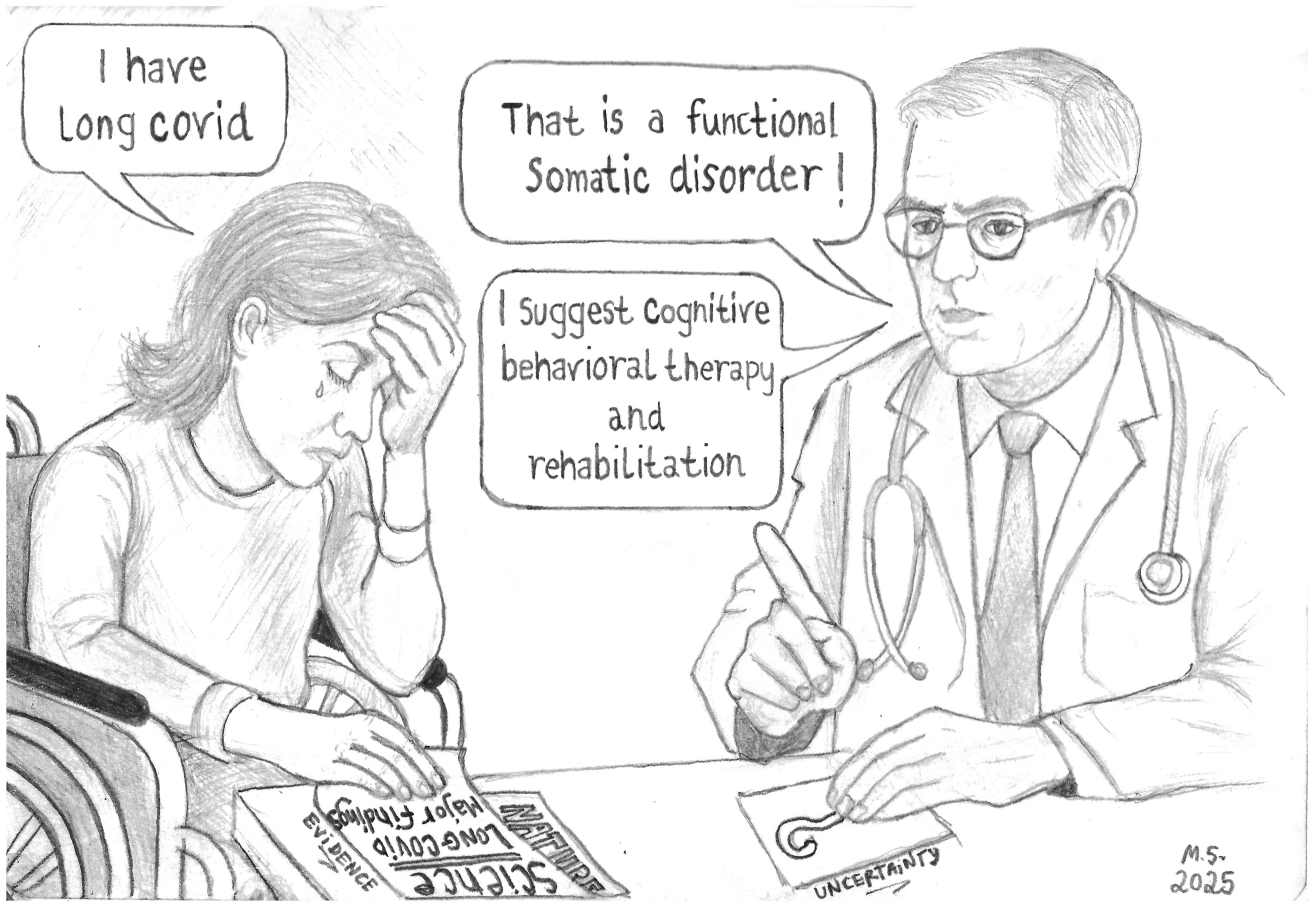
“Evidence and uncertainty.” Sketch inspired by the testimony of a patient suffering from Long COVID for 4 years, upon leaving a consultation where a doctor stated she was suffering from a “Functional Somatic Disorder,” despite showing biomedical evidence. The faces shown in the image are entirely fictional and were generated for illustrative purposes. They do not depict or reproduce any real individuals, and therefore no identifiable person can be recognized.

Conversely, reflexive practice enables clinicians to acknowledge patients' embodied uncertainty, support shared decision-making, and strengthen the therapeutic alliance. Paradoxically, such reflexive practice—presumably central to FSD-oriented care—is absent from frameworks often described as “*functional*,” “*holistic*,” or “*biopsychosocial*” (65). As a result, FSD may engender stigma, adversely affecting patient wellbeing, healthcare experiences, and clinical outcomes (66), consistent with LC patients' testimonies, which highlight that limited clinical reflexivity and epistemic humility can turn uncertainty itself into a source of harm (67) (Figure 1).

## 3 Discussion

LC is heterogeneous both clinically and biomedically, encompassing multiple symptom clusters, trajectories, and levels of impairment that likely reflect partially distinct underlying mechanisms and care needs, thereby underscoring the need for biomedical subclassification. Notably, LC is now widely described as a PAIS (68–73), supported by comprehensive biomedical frameworks that integrate replicated findings across geographically distinct cohorts (74–80) and by emerging interventions targeting underlying pathophysiological mechanisms (51, 81). In this regard, we suggest the article overemphasizes psychosomatic/psychosocial explanations by drawing primarily on a narrow subset of the literature over the broader biomedical and medico-sociological record; evidence from chronic disease research indicates that psychological responses typically reflect the consequences of prolonged illness rather than its primary cause—a pattern also observed in LC (82–86).

Moreover, this subset is then viewed through the lens of FSD. As highlighted in 2.1, FSD's conceptual rationale as well as its application in clinical practice–both solely defined and applied at the symptom level–by design suffer from the limitation of vast heterogeneity, and by implication from a lack of representativeness, translational validity and causal attribution. Subsequently conceptual and practical critique to FSD and similar frameworks like *somatic symptom disorder* (SSD) are frequently abated through what resembles a *motte-and-bailey* pattern (87): shifting from strong claims of functional or psychological etiology to broader, more readily defensible “multifactorial,” “biopsychosocial,” or “stress-related” formulations when challenged (88, 89). Thus, the limited biological components of these diagnoses are overemphasized, while insufficient consideration is given to their lack of specificity, predictive value, and underlying validity, instead centering on non-specific symptoms such as “fatigue.”

These concerns extend to everyday diagnostic and treatment practice, as illustrated by the diagnostic criteria for SSD (90). While criterion A allows for virtually any biopsychosocial factor to account for symptom onset or persistence, the appropriate application of criterion B depends heavily on the clinician's knowledge, reflexivity, and epistemic humility. As a result, ostensibly patient-related observations, such as “excessive thoughts, feelings or behaviors” and “an ongoing high level of anxiety about health or symptoms,” may instead reflect clinician bias or countertransference.^2^ Accordingly, the scientific and clinical use of FSD-like concepts may contribute to diagnostic creep, patient stigmatization, and psychological and physical harm. This need for humility also applies to integrative biopsychosocial or holistic models, which may have value in PAIS if their current scientific and clinical limitations beyond the symptom level are acknowledged and if they are held to the same standards of scrutiny as biomedical evidence, rather than being used as etiological or therapeutic shortcuts in the absence of biomarkers or effective treatments. Acknowledging the vast and growing body of evidence for the biological underpinnings of LC and PAIS, together with their overlap with—and implications for—other diseases (80, 91–93), does neither discount the relevance of psychological or social factors in symptom experience—as in any chronic illness—nor diminish the need for psychological/social support as well as for long-term health outcome monitoring (69, 94, 95). Rather, it reinforces the need for integrative, flexible, and relational care grounded in epistemic humility (67), while acknowledging the history and actuality of iatrogenesis in PAIS (65).

A multifaceted, interdisciplinary clinical approach prioritizing targeted biomedical perspectives should include:

- partnership with patient experts living with emerging illnesses to enhance conceptual clarity,
- rigorous causal inference before mechanistic attribution,
- integration of biomedical data, conceptualizing subclassification and targeted therapies,
- cultivation of reflexivity and epistemic humility (e.g., explicit uncertainty communication, shared decision-making, and iterative reassessment).

## 4 Conclusion

We hope this commentary contributes to a balanced and evidence-based interpretation and management of LC, helping bridge translational gaps, and highlighting the need of biomedical priorities in the sustained collaboration between researchers, clinicians, and patients to advance understanding, diagnosis, and treatment of this evolving condition and other PAIS (96).

## Footnotes

1 Terminology: Psychologizing refers to overemphasizing psychosocial factors at the cost of biomedical ones, albeit causally or at the symptom level. This differs from the modulation of psychological symptom experience or coping, from psychiatric comorbidity and from primary psychiatric presentations in multi-system illnesses.

2 Countertransference refers to clinicians' subconscious emotional, cognitive and behavioral reactions to a patient, based on their own experiences or feelings. This can but need not be a response to a patient's own transference: how his or her history influences how he/she feels about and reacts to a clinician.
